# A mycobacterial disease is associated with the silent mass mortality of the pen shell *Pinna nobilis* along the Tyrrhenian coastline of Italy

**DOI:** 10.1038/s41598-018-37217-y

**Published:** 2019-02-25

**Authors:** F. Carella, S. Aceto, F. Pollaro, A. Miccio, C. Iaria, N. Carrasco, P. Prado, G. De Vico

**Affiliations:** 1 Department of Biology, University of Naples Federico II, MSA, 80126 Naples, Italy; 2 Centro Studi Ecosistemi Mediterranei, Pollica, 84068 Salerno, Italy; 3 Area Marina Protetta di Punta Campanella (AMP Punta Campanella), Massa Lubrense, 80061 Naples, Italy; 4 Department of Veterinary Science, University of Messina, Messina, Italy; 5 IRTA-Sant Carles de la Ràpita, Sant Carles de la Ràpita, Spain

## Abstract

Disease is an increasing threat for marine bivalves worldwide. Recently, a mass mortality event (MME) impacting the bivalve *Pinna nobilis* was detected across a wide geographical area of the Spanish Mediterranean Sea and linked to a haplosporidian parasite. In 2017–2018, mass mortality events affecting the pen shell *Pinna nobilis* were recorded in two different regions of Italy, Campania and Sicily, in the Tyrrhenian Sea (Mediterranean Sea). Histopathological and molecular examinations of specimens showed the presence of *Haplosporidium* sp. in only one specimen in one area. Conversely, in all of the surveyed moribund animals, strong inflammatory lesions at the level of connective tissue surrounding the digestive system and gonads and linked to the presence of intracellular Zhiel-Neelsen-positive bacteria were observed. Molecular analysis of all of the diseased specimens (13) confirmed the presence of a *Mycobacterium*. Blast analysis of the sequences from all of the areas revealed that they were grouped together with the human mycobacterium *M. sherrisii* close to the group including *M. shigaense*, *M. lentiflavum* and *M. simiae*. Based on pathological and molecular findings, it is proposed that a mycobacterial disease is associated with the mortality episodes of *Pinna nobilis*, indicating that, at this time, *Haplosporidium* sp. is not responsible for these events in Campanian and Sicilian waters.

## Introduction

The bivalve pen shell *Pinna nobilis* is an endemic Mediterranean species and among the largest bivalves worldwide, playing an important ecological role for soft bottom communities and contributing to the increase in local biodiversity. This species can reach up to 120 cm in length and a maximum reported age of 27 years^1,2^. The species commonly lives in seagrass fields of *Posidonia oceanica* or *Cymodocea nodosa*^2^ but also in non-vegetated estuarine areas and the soft bottoms of marine lakes^3^. The family of Pinnidae includes two genera (*Pinna* and *Atrina*) with 61 species described worldwide^4^. Different anthropogenic factors have adverse effects on the pen shell lifecycle and distribution, bringing structural and functional alterations in habitats and species physiology. Currently, *P. nobilis* has become a threatened and vulnerable species and is legally protected under Annex II of the Barcelona Convention (SPA/BD Protocol 1995), Annex IV of the EU Habitats Directive (EU Habitats Directive 2007), and the Spanish Catalogue of Threatened Species (Category: Vulnerable, Royal Decree 139/2011).

To date, disease conditions and mortality outbreaks have been reported in marine benthic population worldwide, such as corals, sea urchins, molluscs, sea turtles and marine mammals^5–7^ and have also been described in the Mediterranean Sea^8–10^. Evidence suggests that a combination of predisposing and necessary biotic factors is involved in the disease causation, resulting in a scenario of new emerging complex multifactorial diseases^11^. In fact, these diseases have been often linked to changes in host/pathogen interactions and have been associated with increased water temperature, pathogen distribution and virulence, host reduced immune competence and growth^12,13^. The spread of infectious diseases of molluscs due to pathogens of different types has been intensively described in the last years, especially in farmed species^14,15^, with new outbreaks continuing to be recorded globally, representing a large limitation for the aquaculture industry. Conversely, in wild animal populations, only a few descriptions are present, and these outbreaks can involve keystone species, with consequences for the whole ecosystem.

Recently, mass mortality events of the pen shell *Pinna nobilis* were reported over hundreds of kilometres of the western Mediterranean coast of Spain, except for Catalonia, due to a haplosporidian parasite^16,17^.

In Italy, since 2016, mortality episodes of the pen shell have been described by local SCUBA divers and scientists along the Tyrrhenian coastline and encompassing different regions. In early 2017, mass mortality episodes of *P. nobilis* populations were detected in Campania and Sicily, affecting animals of all sizes and affecting 85–100% in prevalence. The aims of this paper are to unravel the bivalve health status and to define the possible causes of the mortality events involving the pen shell *P. nobilis* in these two geographical areas in late 2017 through May 2018.

## Results

### Mass mortality

**Figure 1:**
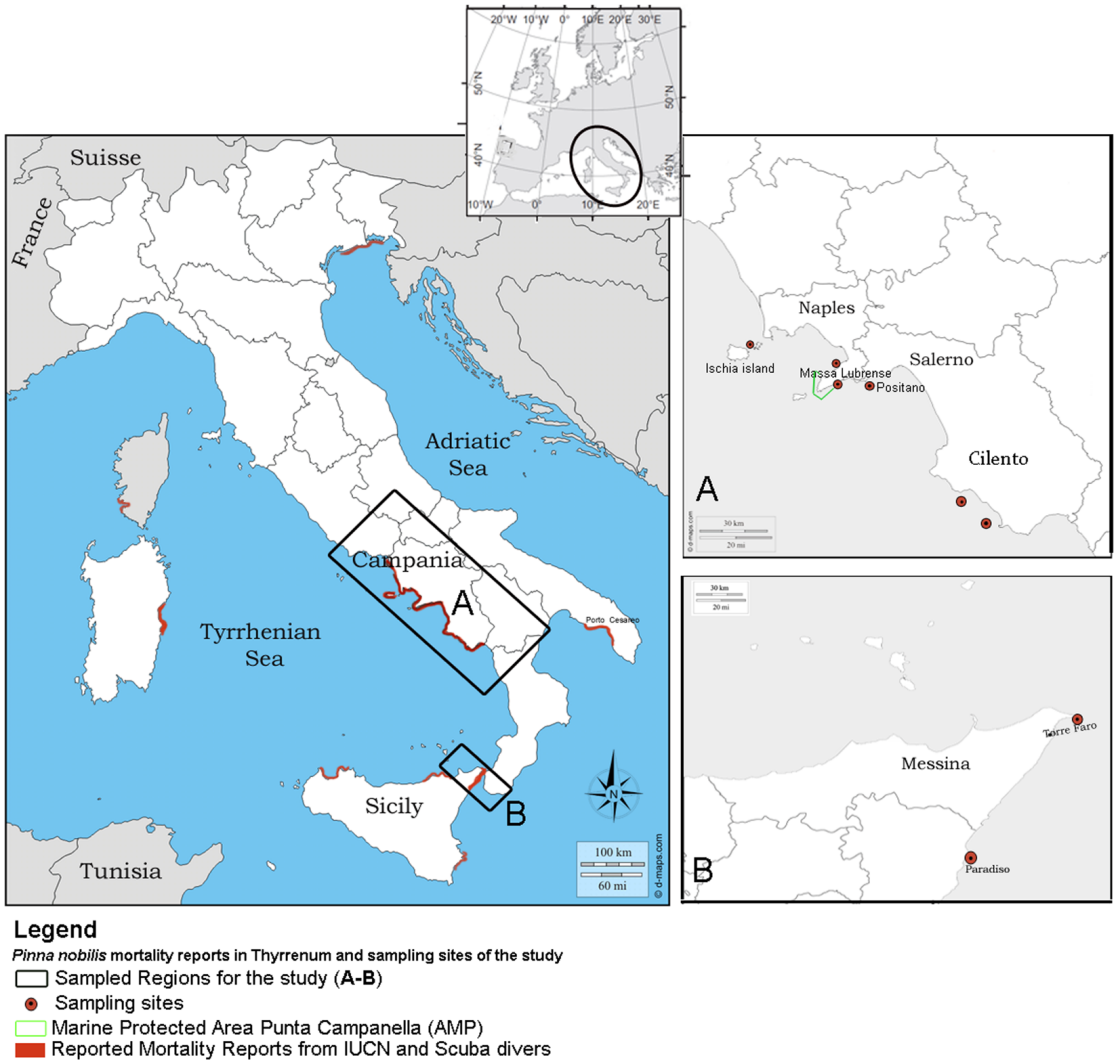
Location map of the two studied Italian regions in Tyrrhenum (**A,B**) (black square) with sampled sites (6 red dots) in Campania and Sicily. Mortality reports from SCUBA divers and IUCN all over Tyrrhenum are underlined in red. Location of the Marine Protected Area- AMP Punta Campanella is denoted by green lines.

Following mortality episodes from different areas of the regions of Campania and Sicily, samples were collected and provided to our laboratory by divers in the Area Marina Protetta (AMP) Punta Campanella from the north-southeast part of the Campania region from December 2017 to May 2018. At the same time, samples were also collected by local fisherman in Sicily (Messina) in May 2018 (Fig. 1). Individuals were placed within iceboxes and transported to the lab for anatomical description, measurement of valve length and dissection of organs.

**Figure 2:**
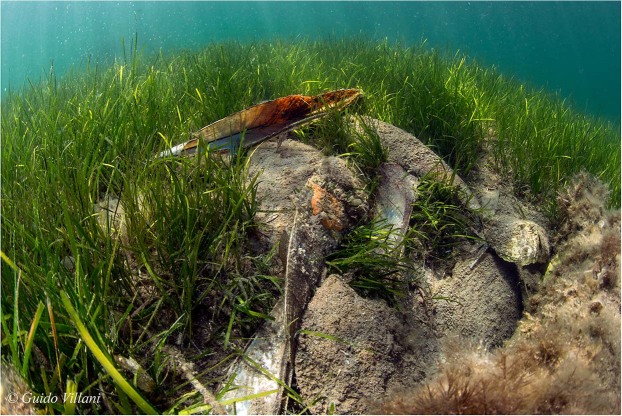
Field observation of dead specimens of *P. nobilis* in Tyrrhenian Sea, Tharros, (OR, Sardinia) at 4 m of depth. Empty and dispersed valves over the phanerogame *Cymodocea nodosa*.

A total of 13 moribund animals of the pen shell *P. nobilis* and 1 specimen of *P. rudis* were collected from two different regions of the Tyrrhenum: Campania and Sicily. At the time of the sampling, in all of the areas, SCUBA divers reported mortality episodes ranging between 80% and 95%, depending on the season and location, and represented by empty valves, as observed in many other part of the Italian Tyrrhenum (Fig. 2). An apparently healthy specimen of *P. nobilis*, which did not show any sign of illness, presenting at external examination well closed valves, strong adductor muscles and good coloration of tissues, was also collected in Campania as a negative control to facilitate disease diagnosis.

Details about animal dimensions, macroscopic lesions and disease diagnosis are reported in Table 1. Collected specimens of shrimp (*P. pinnophylax*) were negative for all of the diagnostic analyses.

**Table 1:** Sample schedules, animal codes and results of PCR analysis of specimens of *P. nobilis*. Positive results are in bold.

Sample date (mo/yr)	Site	Animal code	Animal length (cm)	Histopathology Mycobacterium/Haplosporidium (+/−)	PCR Mycobacterium (+/−)	PCR Haplospordium (+/−)	PCR OsHV-1 (+/−)
12/17	Massa Lubrense 1	ML1-PnDic-17-1	40	**+Myco/**−Haplo	**+** ^a^	−	−
12/17	Massa Lubrense 2	ML2-PnDic-17-1	30	**+Myco/**−Haplo	**+** ^a^	−	−
12/17	Massa Lubrense 2	ML2-PnDic-17-2	38	**+Myco/**−Haplo	**+** ^a^	−	−
12/17	Massa Lubrense 2	ML2-PnDic-17-3	20.8	**+Myco/**−Haplo	**+** ^a^	−	−
12/17	Massa Lubrense 2	ML2-PnDic-17-4	40	**+Myco/**−Haplo	**+** ^a^	−	−
04/18	Ischia	ISPnApr 18-1	46.5	**+Myco/**−Haplo	**+** ^a^	−	−
04/18	Positano	POPn Apr18-1	40.5	**+Myco/**−Haplo	**+** ^a^	−	−
05/18	Cilento 1	CI1Pn May18-1	32.8	**+Myco/**−Haplo	**+** ^a^	−	−
05/18	Cilento 1	CI1Pn May18-2	41.5	**+Myco/**−Haplo	**+** ^a^	−	−
05/18	Cilento 1	CI1Pn May18-3	37	**+Myco/+Haplo**	**+** ^a^	**+** ^c^	−
05/18	Sicily	SICPnMay18-1	54	**+Myco/**−Haplo	**+** ^a^	−	−
05/18	Sicily	SICPnMay18-1	48	**+Myco/**−Haplo	**+** ^a^	−	−
05/18	Sicily	SICPnMay18-2	27	**+Myco/**−Haplo	**+** ^a^	−	−
05/18	Sicily	SICPrMay18-3*	26	−Myco/−Haplo	**−** ^b^	−	−
05/18	Cilento 2	CI2PnMay18-1	38	−Myco/−Haplo	**−** ^b^	−	−

^a^Moribund animal; tissue inflammation with nodules filled by *Ziehl-Neelsen-*positive bacteria.

^b^Non-moribund animal.

^c^Liquid cyst at digestive tissue level.

*Only sampled specimen of *Pinna rudis*.

**Results of the sequence and GenBank accession number are reported in the figures.

### Macroscopic signs of disease

The pen shells did not show any sign of illness, and the only collected individual of *P. rudis* was healthy and negative for all of the diagnostic analyses performed for the different pathogens.

Regarding diseased animals, during collection, anomalous behavioural signs were demonstrated in debilitated specimens, showing difficulty in closing the valves (gaping) or slow responses to touching. The animals also showed retracted mantles or mantles with watery cysts.

On laboratory examination, on the external valves, the animals presented with attached epibionts of different types; among them, polychaetes, bryozoans, red and brown algae, ascidians, sponges and small bivalves were present. All of the specimens contained in the valves the Palaemonidae shrimps *Pontonia pinnophylax*; in 7 cases (53%), there were two per bivalve.

### Microscopic/histopathogical examination

**Figure 3:**
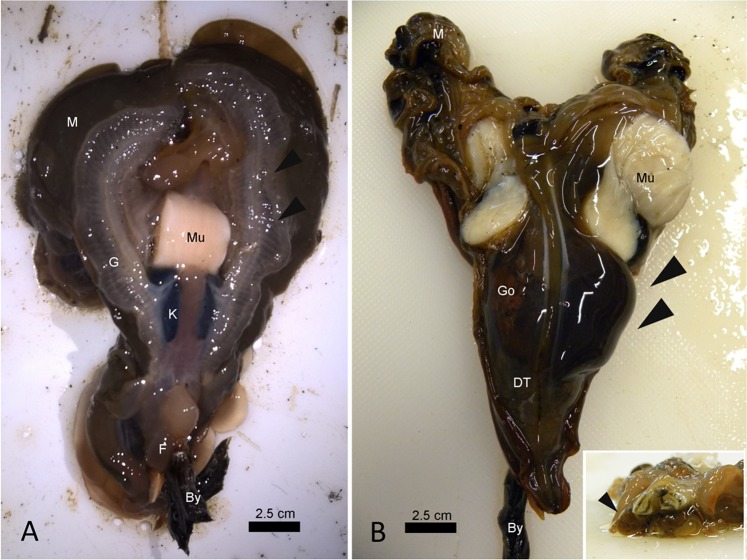
Macroscopic appearance of *Pinna nobilis* sampled from different areas of Campania. (**A**) Gill (G) oedema (arrowheads) in samples from Massa Lubrense 1 in December 2017, posterior view. (**B)** Evident liquid cyst located on the right side of the digestive tissue (DT-arrowheads) and details (insert) of the atrophic and yellowish digestive glands (arrowhead) - frontal view; Go: gonad; Mu: muscle: M: Mantle; By: byssus; K: kidney.

In 9 of the exanimated specimens (69%), gross examination of the bivalves revealed diffuse tissue oedema, mainly visible at the level of the gill and mantle (Table 1, Fig. 3A). Interestingly, in 1 of three examined bivalves from Cilento in 2018 (CI1 PnMay2018–3), a brownish/black cyst-like area was visible at the level of the digestive apparatus (Fig. 3B). Attempts to acquire a cytological smear from the cyst revealed a liquid content, sticky and brownish/yellow in appearance. Once examined, the animal showed an atrophic yellowish/orange digestive gland (Fig. 3B, inset).

**Figure 4:**
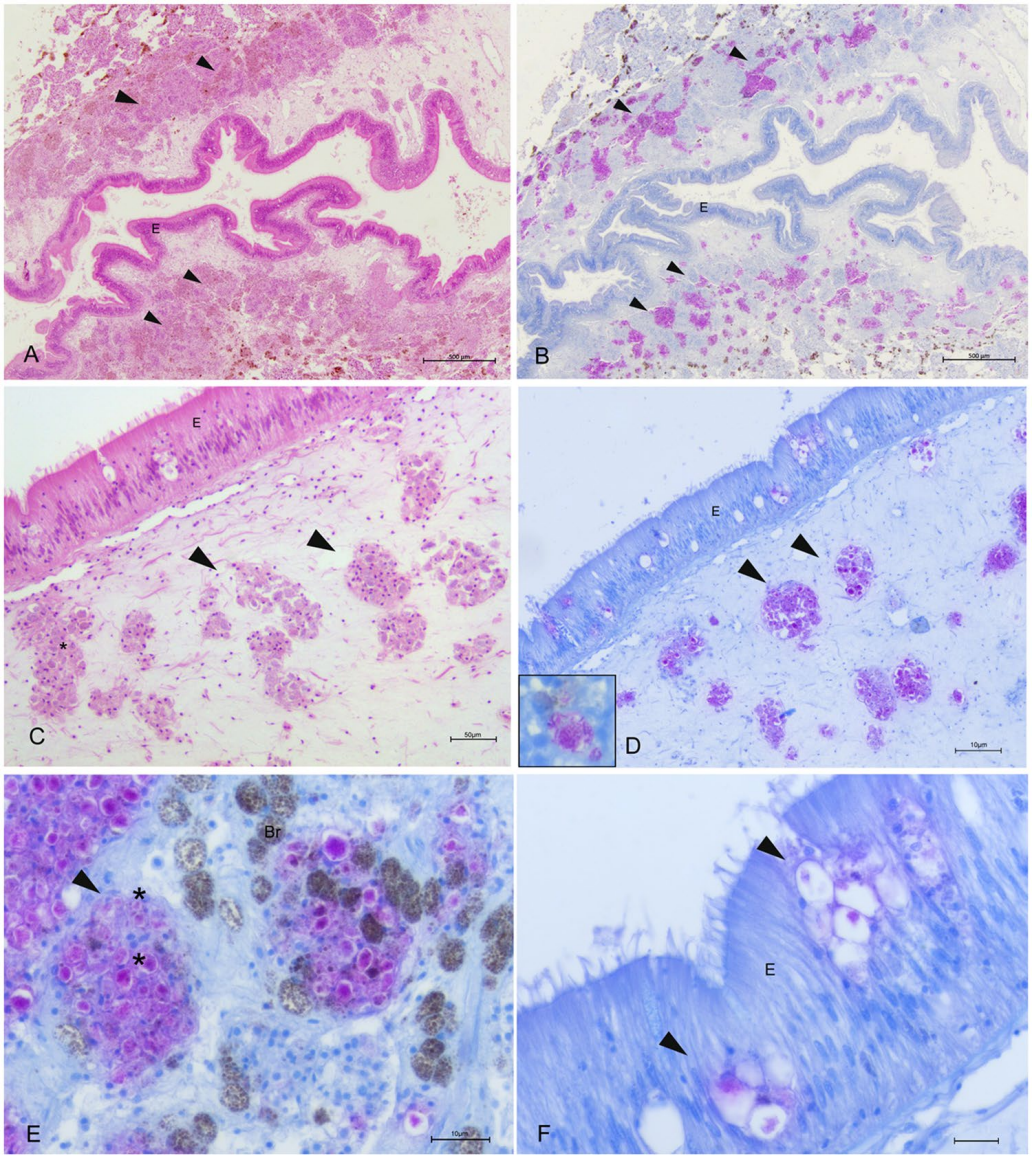
Microscopical observation of Mycobacteria sp. within *Pinna nobilis* immune cells in the bivalve tissues (arrowheads) on H&E (a and c) and Ziehl-Neelsen stain (b,d–f). (**A**,**B**) Connective tissue circumscribing gonads presenting nodules filled with Ziehl-Neelsen-positive bacteria (arrowheads) (**C**,**D**). details of the inflammatory nodules containing bacteria (*) spreading in association with the immune cells and brown cells (Br) (**E**,**F**). presence in the mantle epithelium (**E**) of the mycobacteria (arrowheads); Br: Brown cells.

**Figure 5:**
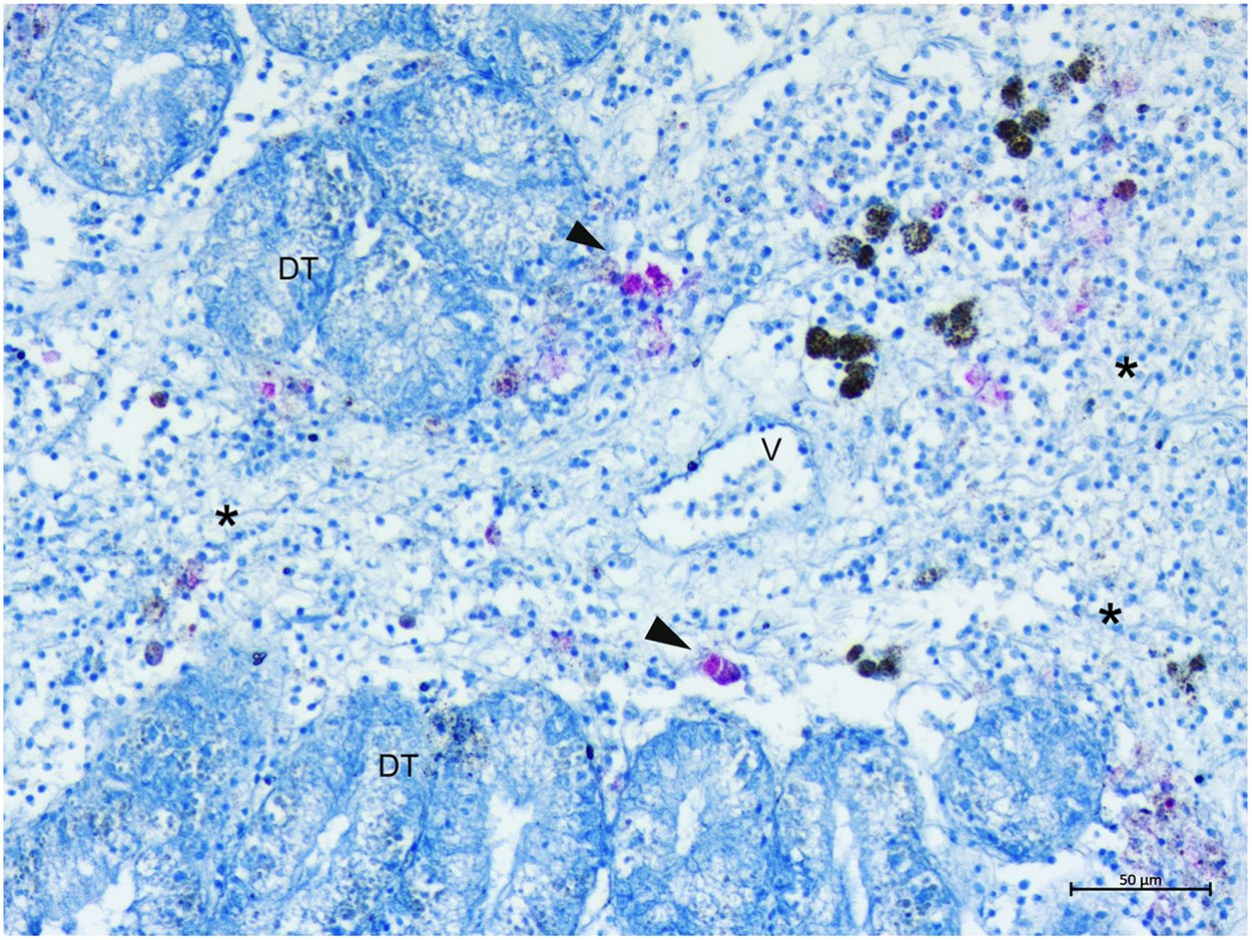
Ziehl-Neelsen-positive mycobacteria (arrowheads) spreading within the digestive gland. **DT:** digestive tubules; V: vessels; *Inflammation.

On light microscopy, all of the specimens showed inflammatory lesions of variable degree; depending on the animals and disease extent, infection seemed primarily present at the connective tissue circumscribing both the gonad and the digestive gland and then in other tissues, such as the mantle and gills (Fig. 4A,B). In two cases, necrosis of digestive tubules and gonadal follicles were also visible. Regarding the inflammatory response, the immune cells involved were of two distinct types resembling those described by^18^ as both hyalinocytes and granulocytes. The inflammatory condition was characterized by large nodular aggregates of the above immune cells, which were filled with long, slightly shaped, acid-fast positive bacteria (Fig. 4C,D). These bacteria-filled immune cells were distributed mainly at the level of connective tissue surrounding the gonads, mantle and digestive tissue, as well as in proximity to the haemolymph vessels forming aggregate-rich regions coupled with Brown cells (Fig. 4E,F). On the other side, infiltrative-type inflammation was instead visible at the digestive tubule level with visible haemocytes filled by mycobacteria around haemolymph vessels (Fig. 5).

**Figure 6:**
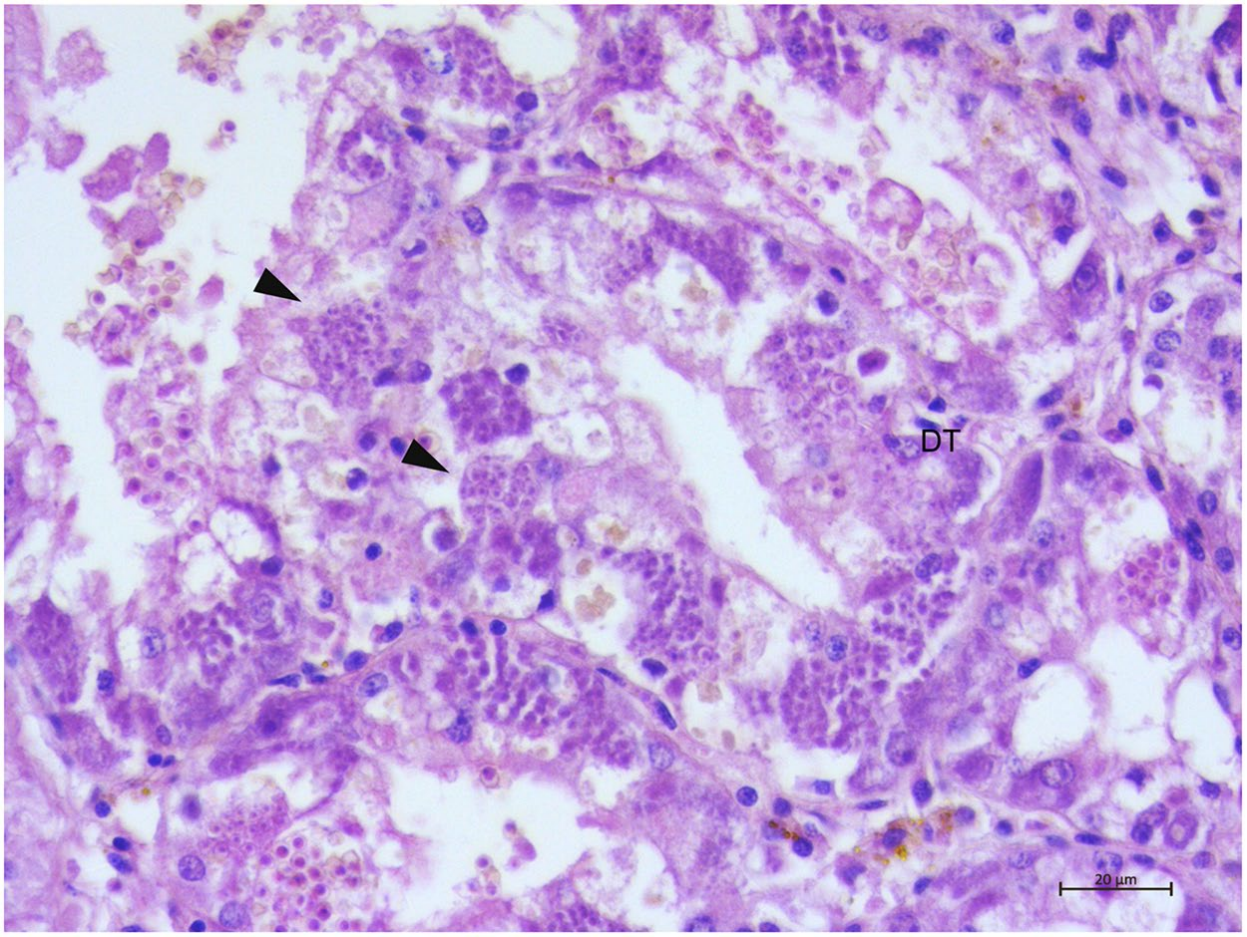
Microscopical observation of the only specimens of *P. nobilis* parasitized by *Haplosporidium* sp. Digestive tissue containing large numbers of the pre-sporulation and sporulation stages (arrowheads); **DT**: digestive tissue. H&E stain.

In the sample from Cilento1 that showed a liquid cyst (CI1PnMay2018-3), both cytology and histopathology showed the presence of different phases of development of a haplosporidian parasite in the digestive tissue. Large numbers of multinucleate stages were disseminated in the digestive tubule epithelia (Fig. 6). Sporogonic stages (sporocysts 20–50 μm in diameter) and acid-fast spores (3.13 ± 0.26 μm of length) were located at the epithelium of the digestive tubules. Sporocyst rupture was seen in the nearby digestive epithelial cells. In the connective tissue, few uni-nucleate stages (2.5 ± 032 μm of length) dispersed with central or slightly eccentric dense nuclei were also observed.

### Ultrastructural studies of the Mycobacterium

**Figure 7:**
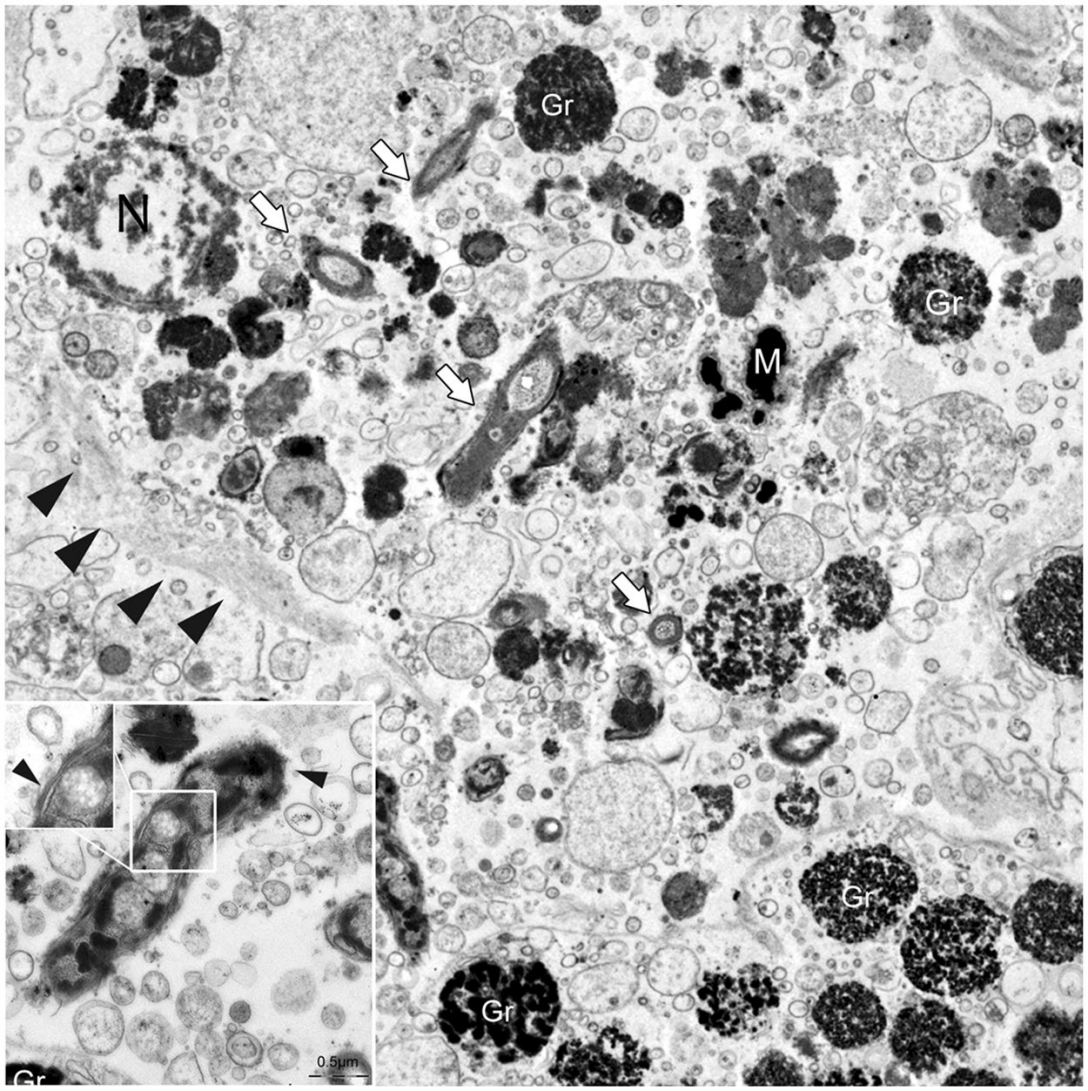
Transmission electron microscopy (TEM) of *Mycobacterium* sp. (white arrows) free in the cytoplasm of immune cells (black arrowheads) with visible nuclei (N). In the inset: details of the rod-shaped *Mycobacterium* with cell walls and containing large vacuoles (arrowhead). **Gr**: immune cell granules; **M**: melanin.

Electron microscopy (EM) was used to further characterize these bacteria in the absence of a cultured strain. Transmission electron microscopy (TEM) performed on three infected individuals was used to assess the features of the bacteria (Fig. 7), allowing for the detection of bacterial shape within the immune cells and showing that they were rod shaped with a diameter of approximately 0.5 μm and a length of 3.5 μm. They contained granules and large vacuoles and were free in the cytoplasm, with no evidence of phago-lysosomal membranes around them (Fig. 7, inset).

### Molecular identification of the pathogen responsible

Considering the pathogens responsible for mortality episodes reported in Italian bivalves and considering the observed *Mycobacterium* sp., three different PCR sets were run for different pathogens: the Herpes virus OsHv-1^19^; haplosporidian parasites^20^; and *Mycobacterium* sp^21^ detection.

**Figure 8:**
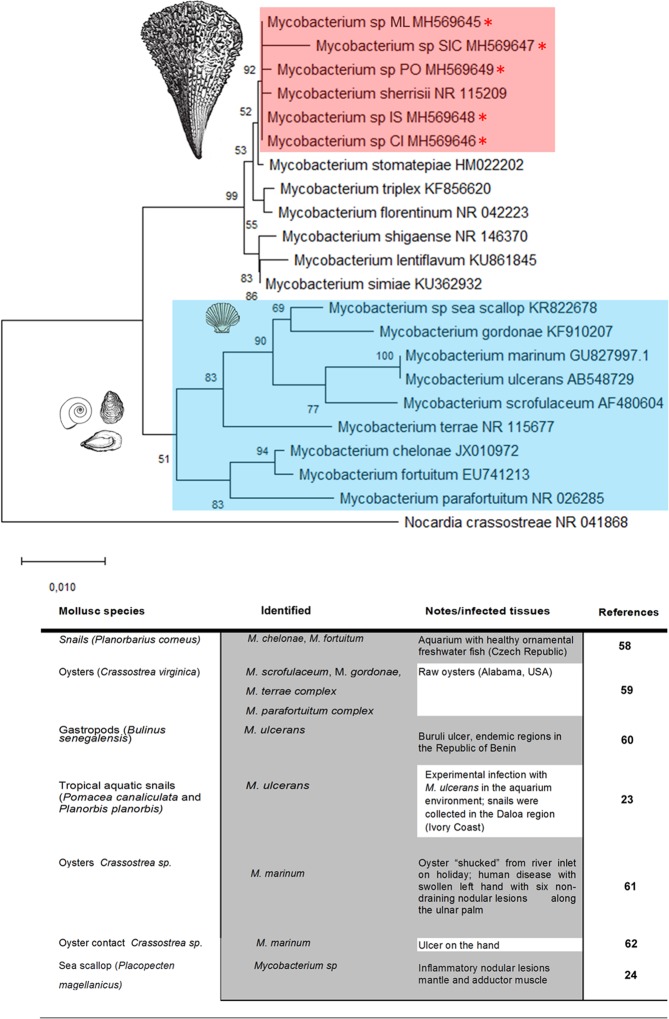
Neighbour-joining tree of the 16S rDNA sequences. The analysis involved 22 nucleotide sequences and 882 positions in the final dataset. The percentages of bootstrap replicates are shown next to the branches. Red asterisks (*) and pink square indicate the sequences obtained in this work (**ML**: Massa Lubrense; **SIC**: Sicily; **PO**: Positano; **IS**: Ischia; **CI**: Cilento). Blue square and table show the reported cases from the literature on mycobacteriosis in mollusc species.

All of the samples were negative for OsHv-1 PCR. In contrast, PCR amplification using the primer pair specific for the 16S ribosomal gene of mycobacteria yielded an amplicon of ∼1000 bp in all of the samples of *P. nobilis* examined apart from the non-moribund specimens from south of Campania (Table 1). BLAST analysis revealed that the nucleotide sequences of these fragments were homologues of the 16S subunit of *Mycobacterium* sp. The *Mycobacterium* sequences isolated from *P. nobilis* were very similar, showing a p-distance ranging from 0 to 0.0069. The neighbour-joining tree constructed from the nucleotide alignment of the sequences obtained in the present study and those of different *Mycobacterium* species downloaded from GenBank are shown in Fig. 8. The sequences of the *Mycobacterium* infecting *P. nobilis* were grouped together with *Mycobacterium sherrisii* and appeared close to the group including *M. shigaense*, *M. lentiflavum* and *M. simiae*. Surprisingly, they are quite divergent from the *Mycobacterium* species previously found in aquatic molluscs (*M. marinum*, *M. ulcerans* and the strain found in sea scallops^22–24^.

**Figure 9:**
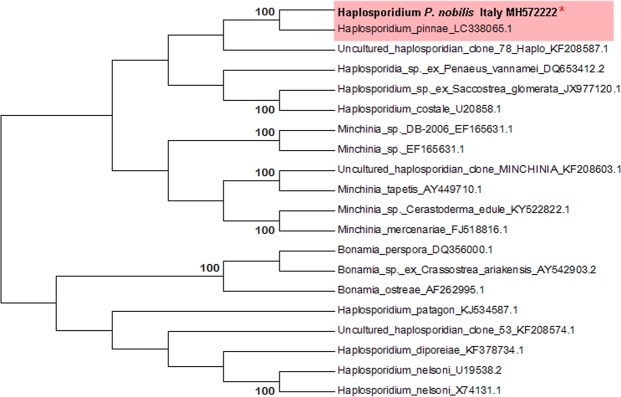
Neighbour-joining tree of the 18S rDNA sequence of haplosporidian parasite. The analysis involved 20 nucleotide sequences in the final dataset. The percentages of bootstrap replicates are shown next to the branches. Red asterisk (*) indicates the sequence obtained in this work.

PCR amplification using the primer pair specific for the haplosporidian 18S ribosomal gene yielded an amplicon of 350 bp for the individual CI1PnMay 2018-3. Blasts showed a similarity of 100% with the *Haplosporidium* sp. identified in Spanish samples of *P. nobilis* (LC338065); moreover, it showed 92% similarity to the 18S small subunit ribosomal RNA of uncultured *Haplosporidium* sp. from environmental samples but was not grouped with any definite haplosporidian species (sequence accession number: MH572222) (Fig. 9). The same individual was positive for the PCR for *Mycobacterium* (Table 1).

## Discussion

In the last decades, numerous episodes of mass mortality events (MMEs) of sessile benthic invertebrates, including ascidians, sponges, anthozoans and bivalves, have been reported in the aquatic environment and many indeed in the Mediterranean Sea. Several factors have contributed to mass-mortality episodes. Many papers have agreed that global warming might be linked to the occurrence of such catastrophic events in the Mediterranean Sea, which could alter the host/pathogen range, due to alteration in host immunocompetence/pathogen virulence and thus modify pathogen transmission rates. In this scenario, opportunistic pathogens are suspected to play an important role, with a modified condition of virulence and pathogenicity^13,16^.

Mycobacteriosis is a serious and generally lethal infectious disease, affecting a wide range of species from human to animals, both in farmed and wild conditions. The Mycobacteriaceae family includes 128 validly published species^25^. With the exception of *Mycobacterium leprae*, the species responsible for tuberculosis in human and animals consists of a group of highly related mycobacterial lineages collectively known as the mycobacterium tuberculosis complex (MTBC)^25^. Finally, MTBC is distinguished from nontuberculous mycobacteria (NTM): free-living organisms that are ubiquitous in the environment and can cause a wide range of mycobacterial infections in humans and animals (more frequently pulmonary infections)^26^.

In the past few years, the occurrence of NTM in both terrestrial and aquatic species has increased^27–30^. In particular, in marine environments, three species have been frequently cited as the main causative agents of infections in fish *M. marinum*, *M. fortuitum* and *M. chelonae* but also several other mycobacterial species, including a number of novel species, have been reported^30–33^. Thus far, mycobacteria in molluscs have only been detected in different gastropod species from the freshwater environment, while in bivalves, frequently oysters of the genus *Crassostrea* have been reported to be vector of infection involved in human disease, and one case was instead reported in the pecten *Placopecten magellanicus* with no human infection^24^ (Fig. 8).

Here, we report a mycobacterial infection affecting the Italian bivalve pen shell *Pinna nobilis* in Campania and Sicily and associated with animal mortality. The found mycobacterium is grouped together with the human mycobacterium *M. sherrisii*, which belongs to a heterogeneous group of mycobacteria part of the group of *Mycobacterium simiae complex*^34^. The first strains of *M. sherrisi* were isolated from HIV-infected patients born in Africa and appeared later in Italy^35^, Argentina^36^ and recently Singapore^37^. Generally, the reports of diseases attributed to NTM have been described in AIDS patients and immune-compromised persons due to cancer, organ transplantation or the administration of immuno-suppressive drugs^38^. Remarkably, many reports have recently observed a progressive pathogenic evolution with cases of NTM also in immunocompetent individuals^38,39^.

Regarding disease pathogenesis and recorded lesions, infection with Mycobacteria typically brings an inflammatory disease condition. The defensive response generally starts with the aggregation of macrophages in the injured foci, with a gradual shift towards an epithelioid cell morphology. As infection progresses, the initial intense pro-inflammatory response observed is regulated by suppressive mediators balancing inflammation. In our cases, mycobacteria were observed within the molluscs’ immune cells, forming typical inflammatory nodular lesions distributed at the level of the connective tissue of the gonads and digestive glands and then spreading to the other organs. Regarding possible pathogenic route, we can speculate by considering the literature on other species of mycobacteria. In vertebrate mycobacteriosis, the general route of infection can be through the digestive, respiratory or congenital (maternal-foetal transmission) system. In the case of *P. nobilis*, the digestive route could be preferential since the main acute lesions were localized at the digestive gland level; from this position, it is likely that the mycobacterium spreads to other organs via haemolymph, as suggested by the presence of haemocytes filled by mycobacteria both within the circulation and around haemolymph vessels. In this sense, this possible pathogenesis is similar to many other types of mycobacteriosis that involve spreading of the mycobacteria via the haematic and lymphatic routes to the entire body, becoming a systemic disease. Previous descriptions of *Mycobacterium* sp. in the Atlantic sea scallop *Placopecten magellanicus* sp^24^ were also represented by an inflammatory response constituted by large nodules with central necrosis but mainly present at the adductor muscle level; haemocyte inflammation, muscular necrosis and mild oedema were also visible in the other tissues, in some cases involving digestive tubules. Phylogenetic analyses showed great differences between this species and the one described in our cases.

With the exception of *M. ulcerans*, *Mycobacteria* are primary intracellular parasites of phagocytes. Phagosomes containing mycobacteria are believed to resist the normal processes of acidification and phago-lysosomal fusion^40–42^, thus promoting bacterial survival^43–45^. However, according to electron microscopy results, the *Mycobacterium* detected in our cases were located in the cytoplasm of immune cells without evidence of phagosome membranes around them. This finding seems to be in accordance with early evidence, suggesting that mycobacteria might eventually escape from phagosomes by translocating to the cytosol, as in the cases of *M. tuberculosis* and *M. marinum*^46^. It has been suggested that cytosolic translocation might reflect a strain-dependent virulence mechanism of pathogenic mycobacteria, conferring to cytosol-preferring strains a gain in virulence function by acquiring resistance to autophagy^47,48^. In this context, *Mycobacterium sherrisii* has been so far associated with several human diseases^35,49^ and only a few cases have been reported in animals, all of them in mammals^50^. To our knowledge, this report is the first concerning evidence for the involvement of *M. sherrisii* in disease outbreaks of aquatic organisms and in a bivalve mollusc. Over thousands of years, mycobacteria have undergone extensive specialization, particularly with vertebrate hosts or specific environmental ecosystems retaining the flexibility to occupy new niches by continually infecting different animals as primary pathogens or opportunists. In our cases, the zoonotic potential of the observed Mycobacterium should be considered and clarified.

Mycobacteria are known to infect a number of aquatic organisms other than fish^28^, surviving and replicating within various hosts^51^, so vectors are potentially present throughout the food web. In many cases, the modality of transmission is not clear. Water and associated biofilms are natural habitats for *Mycobacterium* spp. including *M. marinum*, *M. fortuitum*, and *M. chelonae*, so waterborne transmission seems likely. In our case, the origin and transmission of the Mycobacterium remain unknown, but aquatic environments constitute the natural habitat of *M. simiae* complex.

Disease is the outcome of complex interactions among the host, causative agent(s) and environment. Many causes frequently cooperate to induce diseases (complex of causes), and some of them are necessary (their absence prevents the onset of the effect), while others are predisposing (preparing the ground for the action of the necessary cause)^11^. In this context, it is important to emphasize that the boundaries between pathogen and symbiont can be, in some cases, unfixed, with an alteration in the association, even in evolutionary short periods of time^52^. About haplosporidians, the group includes more than 50 described species, many of them responsible for important diseases in aquatic invertebrates^53^. An haplosporidian parasite was detected across a wide geographic area of the Spanish Mediterranean Sea (western Mediterranean Sea) in early autumn 2016 and was recognized as possibly responsible for the MME of *P. nobilis* population in the area^16,17^. A previous hypothesis reported that the observed *Haplosporidium* was instead a symbiont that modified its relationship with the host following environmental changes, finally leading to the mortality outbreak^17^.

In such a scenario, we consider that data reported in our study drive a hypothesis of a more complex disease pathogenesis involved in the disease outbreak of the bivalve *P. nobilis*, in which two possibly opportunistic pathogens are involved in two different areas of the Mediterranean Sea, suggesting that both are activated by further possible common unidentified causes. Based on our pathological and molecular findings, we are more likely to consider that a mycobacterial disease is associated with the observed mortality episodes, indicating that, at this time, *Haplosporidium* sp. is not responsible for the events in Campanian and Sicilian waters. Further knowledge of the modality of transmission, distribution and source of this pathogens is key to devising methods for the identification and understanding of disease pathogenesis. In our cases, the lack of genomic and proteomic data does not allow us to go beyond the consideration that further studies are needed to clarify the pathogenicity and potential virulence for the reported mycobacteriosis other than its origin and evolution. The present study is especially relevant since it uncovers additional information about the complexity in understanding the multifactorial diseases that we have experienced in recent years in the aquatic environment. Future collaboration among wildlife ecologists, environmental biologists, animal pathologists and related disciplines should be conducted to deeply explore emerging diseases in marine wildlife.

## Methods

### Sampling and light microscopy

The study area in Campania covers a large geographical scale including the main habitat for *P. nobilis* from 5 sites: in December 2017 in Massa Lubrense 1 (40°36′45,7″, 14°19′58,1″) and Massa Lubrense 2 (40°34′57.5″N 14°21′22.6″E); Ischia island, AMP Regno di Nettuno (N 40°42,075′, 013°56, 268′E) and Positano in April 2018 (40°37′3,6″, 14°27′27,1″); and in May 2018 Cilento 1 (39°59′58.59″N, 15°25′40.13″E) and Cilento 2 (40°10′23.9″N 15°05′08.4″E). In Sicily, there were two places: Torre Faro (35°15′50.18″N, 15°38′34.71″E) and Paradiso (38°13′6.91″N, 15°34′4.78″E) (Fig. 1). A permit for collection of *P. nobilis* individuals was issued by the ISPRA - Istituto Superiore per la Protezione e la Ricerca Ambientale (Ref.: *Prot. 25888 5 April 2018*). During the macroscopic evaluation of the individuals, a description of each specimen’s condition was conducted, and the presence of epibionts and valve length were recorded. Animal valves were opened with the help of a blade, and the flesh observed was examined macroscopically, checking for eventual external signs. Impression smears of the digestive glands, gills, mantle and ovaries were obtained, air dried, fixed in absolute ethanol, and stained with May–Grunwald–Giemsa quick stain (Bio Optica, Milan, Italy) for cytological examination. Samples from the digestive glands, mantle, labial palps, gills, gonads and adductor and retractor muscles were fixed in Davidson’s solution for 48 h at room temperature. Pieces of digestive gland and mantle were also preserved in 2.5% glutaraldehyde for TEM examination. Fragments of bivalve tissues and shrimp *P. pinnophylax* were fixed in absolute ethanol for DNA isolation.

*P. nobilis* and *P. pinnophylax* tissue samples were embedded in paraffin blocks and cut to 5 μm with a rotary microtome (Bioptica, Naples, Italy). Tissue sections were deparaffinized, stained with Carazzi haematoxylin and eosin and examined by light microscopy (Zeiss Axioscope A1). Additional staining techniques were also performed: Gram, Mallory Trichrome, Ziehl-Neelsen and PAS-BA (periodic acid Shiff)^54^.

### TEM (transmission electron microscopy)

Five animals from different areas were processed for TEM observation, searching for pathogens of a different nature. From each animal, pieces of digestive gland tissue were placed in 2.5% glutaraldehyde, post-fixed in 2% OsO4, and embedded in Epon. Ultra-thin sections were stained with uranyl acetate and lead citrate and were examined in a JEOL JEM 1010 transmission electron microscope at 80 kV.

### DNA isolation and PCR amplification

DNA was isolated from pieces of different tissues using the Qiagen Blood and Tissue Kit (Qiagen). DNA quality and quantity were checked with a Nanodrop ND-1000 spectrophotometer (Nanodrop Technologies, Inc.).

DNA was amplified by PCR with OsHv-1 primers (OsHVDPFor/OsHVDPRev) by^19^, generic haplosporidian primers (HAPF1- HAPR3)^20^ and primers derived from the 16S rRNA sequence of mycobacteria, mycgen-f (5′-AGAGTTTGATCCTGGCTCAG-3′) mycgen-r (5′-TGCACACAGGCCACAAGGGA-3′), as described by^21^. A positive control was used for OsHv-1 PCR reaction. The PCR was performed in 25 μl of reaction volume containing 1 μl of genomic DNA, 12.5 μl of GoTaq MasterMIX (Promega) at 1x concentration, 6.5 μl of water and 2.5 μl of each primer (10 μM). PCR products were electrophoresed on 2% agarose gels in 1x TAE buffer. The amplified fragments were gel eluted and directly sequenced. Negative controls were included in the PCR reaction.

BLASTN analysis was conducted using the nucleotide sequences obtained in the present study. The sequences were then submitted to GenBank (the accession numbers are listed in Figs 8 and 9).

The pairwise p-distances were calculated among the 16S nucleotide sequences of *Mycobacterium* sp. obtained in the present study and those of different *Mycobacterium* species present in GenBank, selected on the basis of the highest BLASTN score. The analysis also included the 16S sequence of *Mycobacterium* sp. KR822678 isolated from sea scallops^24^ and of *M. marinum* and *M. ulcerans*, which are able to infect marine molluscs^22,23^. The nucleotide alignment was constructed using ClustalW and the neighbour-joining tree was obtained using the Maximum Composite Likelihood model implemented in MEGA X^55–61^ with 1000 bootstrap replicates.
